# Searching for diagnoses and subgroups: a suggestion for criteria

**DOI:** 10.3389/fpsyt.2023.1292917

**Published:** 2024-01-08

**Authors:** Tilman Steinert

**Affiliations:** Klinik für Psychiatrie und Psychotherapie I der Universität Ulm (Weissenau), Ravensburg, Germany

**Keywords:** subgroup, classification, diagnosis, proposal, mental disorders

## Abstract

New subgroups of psychiatric disorders are often claimed. In contrast, classification systems have repeatedly had to abandon established subgroups such as paranoid vs. disorganised and catatonic schizophrenia due to lack of empirical evidence. Four criteria are proposed that should be met to claim valid subgroups: 1. distinct distribution of the defining characteristic between groups; 2. significant differences in variables other than those defining the subgroups cross-sectionally and longitudinally; 3. long-term stability; 4. significant differences between groups in aetiology, pathophysiology, and evidence-based therapy. In contrast to examples from somatic medicine, such as type 1 and type 2 diabetes, few psychiatric disorders meet these requirements.

## Diagnoses and subgroups in the history of psychiatry

“Maybe there is a subgroup…” is a popular conclusion in research articles with inconsistent results. Psychiatrists, as well as physicians in general, like to classify patients into diagnostic categories and suggest further diagnostic subgroups. While the DSM-1 contained 106 diagnostic classifications, the DSM-5 contains 374. The idea that psychiatric classification should refer not only to observed actual symptoms but also to “underlying” disease entities was first developed by the German psychiatrist Karl Kahlbaum (1828–1899) in his textbook (1). Two decades later, Emil Kraepelin (1856–1926) introduced the distinction between “dementia praecox” (since 1911 replaced by Eugen Bleuler’s term schizophrenia) and “manic-depressive psychoses” in the seminal 6th edition of his textbook in 1899 (2). Not only has this distinction strongly influenced all classification systems up to the present day, but since then psychiatrists have been searching for “natural” disease entities in their field, mostly based on assumed distinguishable neurobiological pathways. This is the so-called “medical model” (3, 4). The widely held idea is that such diagnoses represent entities that differ from others in terms of their specific etiology, pathophysiology, and, last but not least, appropriate treatment. With the introduction of criteria-based diagnostics, these classifications have become quite reliable (5), while their validity (as representations of natural entities) has mostly not been proven. Instead, from the perspective of social scientists, the world of mental disorders is characterized by clusters of symptoms rather than clearly distinguishable categories, which has been the source of repeated criticism (6). Nevertheless, a considerable number of subcategories have been introduced into classification systems based on clinical experience and tradition. The strength of subgroup proposals lies in the promise of establishing fairly homogeneous patient groups with similar clinical phenomenology in terms of symptoms and behaviour and, hopefully, specific treatment approaches. The other end of the spectrum of initiatives to restructure psychiatric diagnoses and classifications is theory-driven and increasingly supported by evidence, conceptualising symptoms hierarchically in the Hierarchical Taxonomy of Psychopathology (HiTOP) model (7) and disorders as dimensions in the Research Diagnostic Criteria (RDoC) (8). However, the RDoC show only very weak associations with observed phenotypes (i.e., symptoms and behaviour), and for both approaches any clinical relevance remains a promise. The best known historical example of categorical subgroups is depression with its subdivision into endogenous, neurotic, and reactive depression, with allegedly different etiology, neurobiology, and therapeutic approaches, a path that was followed in research and practice for decades until the late 1990s. As the evidence for the existence of these subgroups remained completely inconsistent, this subdivision had to be abandoned in favour of the current simple severity classification (mild, moderate, severe), which is essentially dimensional rather than categorical in nature. The same has happened with other subgroups. The well-established subgroups of schizophrenia – catatonic, disorganised, paranoid – were finally abandoned in DSM-5 and ICD-11 because empirical research showed that these subgroups overlap strongly and that symptom profiles often change over time. Catatonia, traditionally considered a severe form of schizophrenia, is now classified as a distinct group, as it has been recognised that catatonic symptoms also occur in psychotic disorders, depressive disorders and intoxications, among others (9). In autistic disorders, the division into Asperger’s disorder and pervasive developmental disorder had to be abandoned in DSM-5 and ICD-11 in favour of a (continuous) spectrum (autism spectrum disorder) with comorbidities. Explicitly, the idea of a sharp discontinuity between disorders is no longer maintained in DSM-5.

Thus, on the one hand, we observe a reduction of subcategories in the classification systems due to lack of evidence, moving towards spectrums without the claim of discontinuity, and on the other hand, new research papers still claim to have found “clinical and biological subgroups” (10–12).

## Pragmatic and valid subgroups

Perhaps it is time to define criteria for establishing diagnostic subgroups, free from theoretical (and ideological) assumptions, based on empirical evidence and simple logic, as far as clinical approaches are not congruent with completely dimensional systems as RDoC. First, everyone is free to define subgroups for practical purposes, for whatever reason. For example, subgroups may be defined by height (an interesting example we will return to), health insurance status, medication adherence, presence or absence of certain symptoms, presence or absence of biological markers such as certain genes or autoantibodies, or rural or urban residence. Of course, these are not subgroups in the sense of ‘disease entities’, but they may be helpful for practical reasons. I would suggest calling this type of subgroup pragmatic subgroups. In contrast, subgroups or groups that could represent diagnostic entities should be called valid subgroups.

## Criteria for valid subgroups

Valid subgroups are well known in medicine. A good example that can serve as a reference is the subdivision of diabetes mellitus into type 1 and type 2 diabetes. The distinction between the groups is clear, with very few ambiguous cases. Autoimmune antibodies are present in type 1 and absent in type 2. Moreover, the types differ in aetiology, clinical course, pathophysiological pathways and mechanisms, outcome and therapy. Therefore, the first important criterion to separate valid subgroups or groups is discontinuity, ideally a clear distinction separating the groups along the defining variable. Even a relatively small number of ambiguous cases can get the whole concept into trouble, as can be seen with sex and gender. What does that mean for mental disorders? Most of them seem to be dimensional in their nature and the symptoms, even if categorised categorically in DSM and ICD, are continuous without a clear distinction from normal states, for example considering depressive mood, alcohol consumption, or anxiety. Mental disorders in current diagnostic systems are characterised not only by one such more or less continuous variable or symptom but by a subset of symptoms that are not mutually exclusive with other disorders. A well-known example is substance abuse. A clear distinction cannot be made between substance-induced psychotic disorders and schizophrenia, because substance abuse is common in the latter. This makes substance-induced psychotic disorder questionable as a valid subgroup. Similarly, hypertension would not qualify as an entity, nor would osteoarthritis, which has blurred boundaries with normal ageing processes. In this respect, the problem addressed is not specific to psychiatry, but also to other medical disciplines, albeit to a lesser extent. On the other hand, there is a fairly clear distinction between disorders with and without delusional symptoms. In this respect, depression with psychotic symptoms would belong to psychotic disorders rather than to depressive disorders, which is supported by evidence (13). Also an arbitrary category such as intellectual disability with/without chromosomal aberration would qualify as a category according to this criterion.

Second, the subgroups should differ significantly in other clinically relevant variables than those that defined the subgroup, both cross-sectionally and over time, with little overlap. This is true for the diabetes subtypes, but not for intellectual disability with/without chromosomal abnormality, and therefore such a subgroup is not clinically meaningful. Similarly, schizophrenia with/without family history would be distinguishable subgroups according to criterion 1, but not according to criterion 2. Instead, many substantial differences of this kind are observed between psychotic disorders and anxiety disorders. The separation of these syndromes therefore makes sense not only from a theoretical but also from a clinical point of view. The second criterion, however, is not sufficient to define a valid subgroup without fulfilling criterion 1. This can be illustrated by the example of height. It is possible to define a subgroup of people with a height of >200 cm. This group shows significant differences from the rest of the population in many aspects other than the defining variable, e.g., the proportion of males and of races, and its members have special needs (clothing, space in cars and aeroplanes, etc.) and special advantages (basketball) and disadvantages (jockeys in horse races) in several aspects of life. Apart from a small number of cases, the group assignments remain stable during adulthood. Nevertheless, no one would argue that people >200 cm tall form a valid subgroup. The obvious reason is that the defining characteristic has a continuous distribution and the cut-off is arbitrary. Differences between people with a height of 199 and 201 cm are likely to be very small, if detectable.

Thirdly, valid subgroups representing ‘entities’ must be longitudinally stable. This is true for types of diabetes, intellectual disability with/without chromosomal aberration, but definitely not for schizoaffective psychosis (14), for example. However, subgroups characterised by socio-demographic or historical variables such as educational status could also qualify as subgroups according to criteria 1–3, although they obviously do not represent valid subgroups. A fourth criterion is therefore needed: The subgroups should differ significantly in terms of aetiology, pathophysiology and evidence-based therapy. This criterion is more difficult than the others because it is less free of theoretical assumptions and the aetiology of mental disorders is often controversial. The multifactorial aetiology of most mental disorders makes this criterion difficult to achieve, and some researchers may see it as rarely met beyond Huntington’s Disease. Correspondingly, evidence-based therapies differ much less than we might wish (who is neither psychotic nor suffering from dementia or substance abuse will receive an SSRI combined with CBT according to guidelines). However, anti-NMDA receptor encephalitis as a subset of psychotic disorders may be a good recent example for a valid subgroups fulfilling all criteria. Table 1 shows the proposed criteria.

**Table 1:** Proposed criteria for valid subgroups.

1 Distinct distribution of the defining characteristic between groups
2 Significant differences in variables other than those defining the subgroups cross-sectionally and longitudinally
3 Long-term stability
4 Significant differences between groups in aetiology, pathophysiology, and evidence-based therapy

## Relevance

These are not just academic considerations from the ivory tower. The classification of mental disorders and beliefs about ‘disease entities’ have a strong influence on how psychiatrists think, what they see and do not see, and how they act. Under the paradigm of dementia praecox, no one thought about the recovery and rehabilitation of these patients. As long as we believed that catatonia was a subform of schizophrenia, we ignored catatonic symptoms in paranoid subtypes and depressive disorders. As long as we believed that voice hallucinations were specific to psychotic disorders, we ignored these phenomena in patients with post-traumatic stress disorder and personality disorders, or claimed that they were only ‘pseudo-hallucinations’ until there was ample evidence to the contrary (15, 16), or the patients were re-diagnosed as psychotic and given antipsychotics instead of psychotherapy. The history of psychiatry is full of such errors, not to mention homosexuality.

## Proof of concept

We apply this concept to a classification of animals supposedly found in an ancient Chinese encyclopaedia, according to an essay by the famous Argentinian writer José Luis Borges (1899–1986) (17). Accordingly, animals can be divided into the following groups (a) animals belonging to the emperor, (b) embalmed animals, (c) tamed animals, (d) milk pigs, (e) sirens, (f) fabulous animals, (g) masterless dogs, (h) those belonging to this group, (i) those behaving like quiffs, (j) those drawn with a very fine brush of camel’s hair, (k) etc., (l) those who broke the water jug, (m) those that look like flies from a distance. “The proposed criteria can be applied to all subgroups, although they do not correspond to current biological classifications. Criteria 1–3 are likely to be met for b, e and f, qualifying them as entities, but not for the others. Criterion 4 does not apply.

If these criteria are applied, there will be much more caution in claiming new subtypes. Implicitly, this refers not only to proposals for new subgroups, but also to established “groups” (diagnostic categories) in the ICD and DSM. Ultimately, there may result a clearer separation between real diagnoses (which represent valid entities) and pure classifications that are reliable (but not necessarily valid) for practical purposes. Valid diagnostic categories may shrink to a few dimensional spectra (syndromes) such as autism spectrum, psychotic spectrum, anxiety spectrum, substance abuse spectrum, etc. and a limited, hopefully growing, number of disease entities such as Huntington’s disease or anti-NMDA receptor encephalitis.

## Data availability statement

The original contributions presented in the study are included in the article, further inquiries can be directed to the corresponding author.

## Author contributions

TS: Conceptualization, Writing – original draft, Writing – review & editing.
